# Changes of Coenzyme A and Acetyl-Coenzyme A Concentrations in Rats after a Single-Dose Intraperitoneal Injection of Hepatotoxic Thioacetamide Are Not Consistent with Rapid Recovery

**DOI:** 10.3390/ijms21238918

**Published:** 2020-11-24

**Authors:** Yevgeniya I. Shurubor, Arthur J. L. Cooper, Andrey B. Krasnikov, Elena P. Isakova, Yulia I. Deryabina, M. Flint Beal, Boris F. Krasnikov

**Affiliations:** 1 Center for Strategic Planning and Management of Medical and Biological Health Risks, Federal Medical-Biological Agency of The Russian Federation, 119121 Moscow, Russia; EShurubor@cspmz.ru; 2 Department of Biochemistry and Molecular Biology, New York Medical College, Valhalla, NY 10595, USA; 3 Independent Researcher, 24 Ivana Babushkina St., 117292 Moscow, Russia; kr-ab@yandex.ru; 4 Bach Institute of Biochemistry, Research Center of Biotechnology of the Russian Academy of Sciences, 119071 Moscow, Russia; elen_iss@mail.ru (E.P.I.); yul_der@mail.ru (Y.I.D.); 5 Department of Neurology, The Brain and Mind Research Institute, Weill Cornell Medical College, New York, NY 10021, USA; fbeal@med.cornell.edu

**Keywords:** acetyl-coenzyme A, coenzyme A, hepatic encephalopathy, high performance liquid chromatography, thioacetamide

## Abstract

Small biomolecules, such as coenzyme A (CoA) and acetyl coenzyme A (acetyl-CoA), play vital roles in the regulation of cellular energy metabolism. In this paper, we evaluated the delayed effect of the potent hepatotoxin thioacetamide (TAA) on the concentrations of CoA and acetyl-CoA in plasma and in different rat tissues. Administration of TAA negatively affects liver function and leads to the development of hepatic encephalopathy (HE). In our experiments, rats were administered a single intraperitoneal injection of TAA at doses of 200, 400, or 600 mg/kg. Plasma, liver, kidney, and brain samples were collected six days after the TAA administration, a period that has been suggested to allow for restoration of liver function. The concentrations of CoA and acetyl-CoA in the group of rats exposed to different doses of TAA were compared to those observed in healthy rats. The results obtained indicate that even a single administration of TAA to rats is sufficient to alter the physiological balance of CoA and acetyl-CoA in the plasma and tissues of rats for an extended period of time. The initial concentrations of CoA and acetyl-CoA were not restored even after the completion of the liver regeneration process.

## 1. Introduction

### 1.1. CoA and Acetyl CoA

Coenzyme A (CoA) and its thioester, acetyl-CoA, are vital biomolecules that play an essential role in regulating metabolism and supplying cells with energy. The study of CoA and a number of its thioesters has focused on understanding the role of these coenzymes in the regulation of cellular metabolism under both normal and pathological conditions.

By participating in the reactions of oxidative metabolism and biosynthesis, CoA is an essential cofactor for biochemical processes in the cell. The metabolism of CoA and acetyl-CoA is strictly regulated in the cell and optimized to stabilize various cellular functions. A deeper understanding and assessment of the possible mechanisms of cell regulation is possible by monitoring the metabolic fluxes of CoA and its thioester derivatives [1]. Acetyl-CoA serves as a bridge, linking together glycolysis, β-oxidation of fatty acids, synthesis of fatty acids, and the tricarboxylic acid (TCA) cycle (Krebs cycle) in mitochondria. An important function of acetyl-CoA in the cell is to deliver the acetyl group into the TCA cycle for its subsequent oxidation and energy production. In addition, acetyl-CoA is responsible for many biologically important acetylation reactions.

The balance in the cell between CoA and acetyl-CoA is of crucial importance for assessing the cells’ functional (normal or pathological) state. As mentioned above, acetyl-CoA is important for fatty acid and glucose oxidation. In addition, carbon from ketogenic amino acids (lysine and leucine) can be converted directly into the acetyl moiety of acetyl-CoA, followed by entry of the acetyl carbon into the TCA cycle. The rate of formation of acetyl-CoA is closely related to the working of the TCA cycle itself, which makes it possible to generate energy in accordance with the immediate needs of the cell. This mechanism is reflected in the regulation of acetyl-CoA production relative to its metabolism, which can be expressed by the acetyl-CoA/CoA ratio in the cell mitochondria [2,3,4]. The metabolism of the acetyl moiety of acetyl-CoA leads to the production of a number of metabolites necessary for normal cell functioning, such as the neurotransmitter acetylcholine, which is reduced in brain neurons in the early stages of Alzheimer disease (AD) [5].

An increase in the mitochondrial acetyl-CoA/CoA ratio, for example, under conditions of low energy consumption, leads to feedback and inhibition of the oxidation of fatty acids and glucose. The main site of inhibition of carbohydrate oxidation is at the level of the pyruvate dehydrogenase (PDH) component of the pyruvate dehydrogenase complex (PDHC). PDHC plays a major role in maintaining adequate levels of acetyl-CoA in the mitochondrial compartment of neurons and presumably other cell types. PDHC inhibitors and activators cause, respectively, a decrease or increase in the level of acetyl-CoA in the mitochondrial compartments [6,7]. The transport of acetyl-CoA from mitochondrial to extramitochondrial compartments depends on the rate of its synthesis by PDHC and the capacity of transport systems in the mitochondrial membranes [8]. Studies carried out on purified bovine kidney and heart PDHC showed that acetyl-CoA and NADH stimulate the activity of PDH kinase (which in turn inhibits PDH), while CoA and NAD^+^ inhibit its activity [9]. Thus, the activity of PDHC decreases with an increase in the molar ratios of acetyl-CoA/CoA and/or NADH/NAD^+^ [10]. This regulation of PDHC has been confirmed in many studies. For example, in fat cells, a 10-fold decrease in the acetyl-CoA/CoA ratio was shown to be accompanied by a 5-fold increase in PDHC activity [11].

As noted above, an increase in the acetyl-CoA/CoA ratio may be accompanied not only by direct inhibition of PDH by acetyl-CoA, but also by PDH-kinase activation. Due to the fact that PDH-kinase phosphorylates inhibits PDH, there is a decrease in carbohydrates oxidation when acetyl-CoA concentrations are increased [2,3]. It was also previously noted that when there is a need to synthesize fatty acids, acetyl-CoA carboxylase is activated by physiological concentrations of CoA [12].

The acetyl-CoA/CoA ratio is not only an important regulator of metabolic reactions in the cell, but also reflects the complex regulatory system of the apoptotic caspase-2 protease (CASP2). This ratio is important in induction of programmed cell death processes, including caspase-dependent apoptosis or regulated necrosis. For example, a decreased acetyl-CoA/CoA ratio reflects the ability of cells to survive through mechanisms that affect CASP2 activity [10].

Previously it was reported that various conditions such as fasting, administration of hormones, drugs, and alcohol, as well as diabetes or the occurrence of various types of tumors can drastically change the content of CoA and affect its homeostasis in tissues [13]. The cellular levels of acetyl-CoA may also differ significantly under physiological and pathological conditions. Thus, the concentrations of these coenzymes are highly sensitive to various external stimuli such as stress, hormone intake, neuroprotective agents, or specific nutrients [14]. However, it is still not clear whether metabolic changes affect the level of CoA in the cell, or changes in the level of CoA lead to an abnormal state in the cell.

CoA plays a crucial role in the metabolism and normal functioning of the nervous system. On the one hand, dysfunction of CoA synthesis often leads to the accumulation of iron in certain areas of the brain that are associated with neurodegeneration [15], while the exact role of CoA in the onset and development of neurodegenerative processes is not yet fully understood [16]. On the other hand, it was recently reported that in cell culture and animal models of age-related neurodegeneration and AD, an increased level of acetyl-CoA due to inhibition of acetyl-CoA carboxylase 1 results in neuroprotection [17].

In light of the above discussion, it is apparent that knowledge concerning the optimal level of CoA content in various tissues or organs, the size of these pools under physiological and/or pathological conditions, as well as an understanding of the possible mechanisms that affect the level of CoA in a cell, can help in creating regimens intended to normalize the function of the TCA cycle in certain types of diseases.

### 1.2. Development of Pathophysiology in Rats and Recovery after TAA Intoxication

This article discusses the changes in concentration of CoA and acetyl CoA in various organs of physiologically healthy rats and in rats that have been exposed to a potent hepatotoxin, namely thioacetamide (TAA).

TAA is an organosulfur compound that is strongly carcinogenic and toxic to many organs, leading to acute liver damage and to the development of hepatic encephalopathy (HE) [18,19,20,21]. When rat hepatocytes are exposed to TAA, it is rapidly metabolized to thioacetamide-*S*-oxide and acetamide. In hepatocytes in culture, thioacetamide *S*-oxide binds to macromolecules, changing both the permeability of cell membranes and the uptake of Ca^2+^, increasing the volume of the nucleus, and inhibiting mitochondrial activity [18]. Moreover, in a mouse model of TAA intoxication, it was shown that TAA not only generates significant liver injury, but also neurological decline, an elevation of circulating ammonia concentrations, cerebral edema, and neuroinflammation [22]. TAA administration also causes changes in the blood-brain barrier (BBB) permeability. The authors of this study suggested that the change in the BBB permeability induced by TAA is not likely due to a direct effect on brain endothelial cells [22]. In animals administered TAA, liver necrosis ultimately ensues [19,20,21]. TAA at high or repeated doses can be lethal.

The study of liver necrosis due to TAA intoxication in rats and its subsequent regeneration showed that the maximum damage to liver cells occurs within the first 24 h after a single exposure to the toxin. Thus, injection of TAA into Wistar rats led to a significant increase in markers of liver disease, DNA fragmentation in hepatocytes, and a marked change in liver morphology [19]. The restoration of liver cells to the “normal” adult state was noted as early as 72 h after TAA intoxication [20]. However, despite the claimed recovery of liver cells in rats as early as 72 h after exposure to TAA, the level of some metabolites was still different from the level of those metabolites in healthy rats [20].

The molecular mechanisms that cause liver damage as a result of TAA intoxication, or its recovery from intoxication, are still not fully understood. Nevertheless, it is generally assumed that intoxication occurs in large part through changes in oxidative homeostasis, energy production, and inhibition of the mitochondrial respiratory chain [21].

The development of liver disease is often associated with an increase in the level of ammonia in the blood and brain [23]. Many enzymes are involved in brain ammonia generation, including glutaminase and glutamate dehydrogenase, but only one enzyme is important for the ammonia removal, namely glutamine synthetase, and its activity may become limiting in HE, allowing ammonia to reach neurotoxic levels [24,25].

A number of studies have shown that hyperammonemia results in decreased cerebral energy metabolism, decreased oxygen consumption, decreased glucose catabolism, increased glycolysis and decreased productivity of the TCA cycle [26,27,28,29,30]. It was previously suggested that the increased ammonia production in HE is associated with increased reductive amination of α-ketoglutarate (α-KG) to glutamate catalyzed by glutamate dehydrogenase (GDH), thereby lowering the concentration of α-KG and slowing the TCA cycle [31]. However, it was later shown that this hypothesis is incorrect. GDH in the brain catalyzes oxidation of glutamate (even during hyperammonemia) rather than reductive amination [25] (see the discussion in ref [32]). However, ammonia has an inhibitory effect on a key TCA cycle component, namely, the α-ketoglutarate dehydrogenase complex (KGDHC) [25,33].

It has also been shown that acute liver failure (ALF) is accompanied by increased production of lactate in the brain due to inhibition of the PDHC, which also leads to a decrease in oxidative metabolism [24,34]. Elevated blood and brain lactate levels have traditionally been considered to be a marker of energy imbalance [34,35].

It is also likely that an increase in lactate levels during HE is due to an increase in pyruvate production as a result of increased glycolysis and the subsequent inability of pyruvate to enter the TCA cycle in the form of acetyl-CoA due to partial PDHC inhibition and/or to increased lactate dehydrogenase (LDH) activity (possibly as a result in part of an increased cytosolic NADH/NAD^+^ ratio) [36]. In this context, the oxidation of pyruvate is inhibited by ammonia in isolated rat liver mitochondria [37]. When respiration was inhibited by ammonia, an increase in the remaining pyruvate was noted [37]. Inhibition of PDHC by ammonia has also been demonstrated in a rat model of acute HE [34]. This can lead to a decrease in the rate of the TCA cycle and have a negative impact on the electron transport chain and subsequent ATP production. In summary, there is no doubt that, despite some controversies, liver disease and severe hyperammonemia decrease brain energy metabolism [24,25,33,34,38].

The liver is a major regulator of nitrogen metabolism. Surprisingly, however, despite the well-documented morphological and physiological changes produced by TAA, administration of this toxin to rats was shown to result in little change of the glutaminase II pathway (glutamine + α-keto acid → α-ketoglutarate + amino acid + ammonia) in various organs (manuscript in preparation). Nevertheless, we suggest that other biochemical pathways may be severely affected by TAA administration. To this end, in this article, we present the results of monitoring CoA and acetyl-CoA in the blood plasma and organs of rats (liver, kidneys, and brain) obtained after a prolonged recovery period (6 days) from TAA intoxication.

## 2. Materials and Methods

### 2.1. Chemicals

All solutions were prepared in Millipore water (Milli-Q system, Billerica, MA, USA). Analytical grade dibasic potassium phosphate ultra-pure powder reagent, aqueous solutions (72%) of perchloric acid (PCA) and high performance liquid chromatography (HPLC) grade acetonitrile were obtained from JT Baker (Phillipsburg, NJ, USA); the trilithium salt of acetyl-CoA, the sodium salt hydrate of CoA, sodium acetate, monosodium phosphate, 85% aqueous phosphoric acid, and the Bio-Rad DC™ protein assay kit were obtained from Bio-Rad (Hercules, CA, USA). All chemicals were of HPLC grade and used without further purification. Nylon membrane filters with a diameter of 47 mm and a pore size of 0.2 µm that were used for mobile phase filtering and degassing were obtained from Pall Life Science (Port Washington, NY, USA).

### 2.2. Standards

Standard stock solutions of CoA and acetyl-CoA (1–10 mM) were prepared in deionized water. These standard stock solutions were stable at −80 °C for a minimum of two years. Sets of mixed CoA and acetyl-CoA were obtained by serial dilutions of stock solutions of the standards in 5% aqueous PCA.

### 2.3. HPLC System

Our HPLC-based method for CoA and acetyl-CoA measurements in tissues and plasma has been described in detail previously [39]. The method was validated by using selected enzyme treatments to remove either acetyl-CoA or CoA from the homogenate. Such treatment resulted in complete removal of the peaks, indicating that no metabolites in the biological preparations interfered with the assay of these compounds [39].

Briefly, for the analysis of metabolites of interest, we used Waters instrumentations (Milford, MA, USA), including a 1525 binary pump, a 2707 autosampler with a pre-cooled platform (4 °C), and a 2489 UV/VIS detector. Data collection and data analysis were managed by Breeze2 software (Waters Corp, Milford, MA, USA) installed on a Dell computer. The wavelength for UV detection of CoA and acetyl-CoA was set at 259 nm.

Separation of CoA from acetyl-CoA was achieved using an RP-C18 analytical column (150 × 3 mm, 3 µm particle size, 120 Å pore size PN# 70-0636, ESA Inc./now Dionex & Thermo Fisher Scientific Company, Bedford, MA, USA), equipped with a Phenomenex Security Guard column cartridge C18 (4 × 2 mm, PN# AJ0-4286, Torrance, CA, USA). The flow rate was 0.5 mL/min, and the injection volume was 30 µl. Under these conditions, CoA and acetyl-CoA elute at 3.8 and 7.8 min, respectively. The run time was completed within 12 min without any additional time for column re-equilibration between the HPLC runs. The analytical and guard columns were maintained at room temperature (22 °C).

### 2.4. Mobile Phase

The initial buffer for mobile phase separation of CoA and acetyl-CoA contained 100 mM monosodium phosphate and 75 mM sodium acetate, adjusted to pH 4.6 with concentrated phosphoric acid. The final mobile phase consisted of 94 parts of initial buffer and 6 parts of acetonitrile (*v*/*v*).

### 2.5. Preparation of Biological Samples for HPLC Analysis

#### 2.5.1. Rat Tissues

The tissue samples were obtained from female outbred Wistar rats (130–140 g weight). All experiments were performed in accordance with the Guidance on the Operation of the Animals (Scientific Procedures) Act 1986 (United Kingdom) and associated guidelines (The animal protocol was approved by the Supervisory Board of the Bach Institute of Biochemistry and the Biotechnology Research Center of the Russian Academy of Sciences, Moscow (23 November 2014). Written informed consent was obtained from all participants). The control group (*n* = 3) was injected intraperitoneally with saline solution only. Three other groups of rats were intraperitoneally injected with different single doses of TAA, 200 mg/kg (*n* = 3), 400 mg/kg (*n* = 6), and 600 mg/kg (*n* = 4). During the first several hours after TAA injection, the rats moderately reduced their food intake, and their physical activity was markedly reduced. However, after 1–2 days, their activity returned to normal and they regained their appetite. All animals were sacrificed by decapitation 6 days after the injection. Plasma, brain, liver, and kidneys were rapidly collected and immediately frozen in liquid nitrogen. The samples were then shipped on dry ice from Moscow to the Weill Medical College of Cornell University prior to HPLC analysis.

For the preparation of the samples for CoA and acetyl-CoA analysis, ice-cold aqueous 5% PCA solutions containing 50 µM dithiothreitol (DTT, PCA-DTT) were used. PCA-DTT solution (200 µL) was added to the frozen brain, liver, or kidney tissue sample (10–20 mg), followed by thorough vortexing, and homogenization on ice by brief sonication for 12 s at amplitude 20%. After further incubation of the homogenate on ice for 10 min, it was centrifuged at 14,000 *g* for 20 min at 4 °C. The pellet was saved for later protein analysis. The supernatant was transferred into a clean Eppendorf tube and centrifuged again under the same conditions. The clear supernatant was carefully transferred to an HPLC vial for direct injection into the HPLC system.

The concentrations of CoA and acetyl-CoA in the sample were calculated as nmol/mg of protein using the corresponding calibration curves.

#### 2.5.2. Rat Plasma

Each rat plasma sample (50 µL) for CoA and acetyl CoA analysis was added to a tube containing 250 µL of ice-cold PCA-DTT solution. The acid-treated extract was vortexed, centrifuged, and processed as described above.

The concentrations of metabolites in the plasma samples were calculated as nM using the corresponding calibration curves.

### 2.6. Protein Analysis

For protein measurements a standard Bio-Rad DC™ protein assay kit (Cat#500–0113, 500–0114, 500–1115) was used. The pellets obtained from deproteinized tissue/plasma samples were dissolved by brief (10 s) vortexing in 0.5 mL of 0.1 M NaOH, followed by a six-fold dilution of the suspension in deionized water. Protein concentration was measured spectrophotometrically, using a SpectraMax M5 platereader (Molecular Devises, Sunnyvale, CA, USA) set at a wavelength of 670 nm (bovine serum albumin was used as a standard).

### 2.7. Data and Statistical Analysis

Data are presented as the Average ± Standard Deviation (AVG ± STDEV).

The choice of the statistical method of analysis is due to the presence of the following circumstances: A small and unequal sample size in groups of experimental animals, as well as to their large individual variability. The analysis was aimed at investigating the presence of a possible relationship between quantitative (concentration of coenzymes CoA and acetyl-CoA in samples) and qualitative (variety of biological samples and doses of TAA administered at 200, 400, and 600 mg/kg) experimental parameters. The sets of animals consisted of four test groups with between three to six rats in a group. For each animal, eight measurements were made (two metabolites in four types of biological samples: Homogenates of liver, kidney, and brain tissues, and blood plasma).

For the dataset obtained, a total of 48 hypotheses were created: 24 independent hypotheses regarding the absence of the effect of the TAA dose versus control (two metabolites, three TAA-induced groups, and four types of biological samples) and 24 independent hypotheses regarding the absence of differences between the experimental groups (two metabolites, three pairwise comparisons of TAA-induced groups, and four types of biological samples).

Multiple hypothesis testing for a preselected significance level of alpha may yield a number of false negative null hypotheses. Therefore, to manage the probability of a False Discovery Rate (FDR), we relied on the Benjamini–Hochberg procedure, and to narrow the search area, we used variance analysis and the Tukey Honestly Significant Difference (HSD) method to highlight the most distinct groups.

## 3. Results

### Concentration of CoA and Acetyl-CoA in Rat Tissues and Plasma

Monitoring the levels of CoA and acetyl-CoA in plasma and tissues of animals is a key to understanding the processes occurring in the cell in terms of its energy homeostasis. According to previously obtained data, the levels of CoA and acetyl-CoA in cells are very sensitive to the effects of endogenous and exogenous stimuli and vary significantly depending on the chosen study model, tissue type, or cell culture [14,39].

To monitor changes in the levels of CoA and acetyl-CoA in the body as a result of pathogenesis, we used blood plasma and tissues of healthy rats, as well as rats that had undergone intoxication with the potent hepatotoxin TAA. The concentrations of both coenzymes in the samples from control rats and from rats that received single injections of TAA at doses of 200–600 mg/kg body weight are presented in Table 1. Plasma and tissue samples of rats were obtained 6 days after the administration of various doses of TAA, which, according to a previous publication [20], should correspond to the complete recovery of liver cells of animals after a single exposure to this markedly toxic compound.

Among the rat tissues investigated, the highest concentration of CoA was found in the liver (Table 1). In the kidneys and brain, the concentration of CoA was, respectively, 4–5 and 10–11 times lower than that in rat liver. Relatively large differences in the content of CoA in tissues were found in the control group of rats, but with an increase in the dose of toxin, these differences were less evident.

The distribution pattern of acetyl-CoA in the liver, kidney, and brain of rats was different from that of CoA. Among the tissues investigated, acetyl-CoA was of highest concentration in the liver, followed by the brain; only small amounts of acetyl-CoA were found in the kidney. The content of acetyl-CoA in the rat brain was 5–10 times lower than that in the liver. The maximum difference in the concentrations of this metabolite in the brain relative to that in the liver was also characteristic of the control group of rats. The individual variability in the content of acetyl-CoA in rat kidneys was even higher than that in the brain. In the kidneys of the control group of rats, the level of acetyl-CoA was almost 60-times lower than that in the liver, while in animals that underwent TAA intoxication, this difference was only 11–13 times lower.

**Figure 1:**
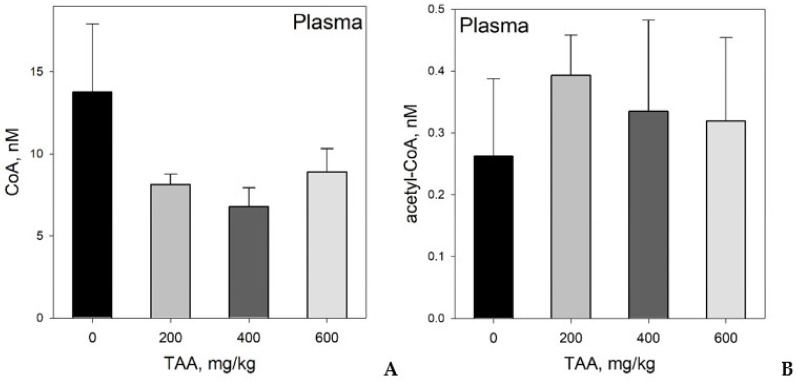
(**A**) Distribution of CoA and (**B**) acetyl-CoA in blood plasma of rats administered different doses of TAA. Data are given as AVG ± STDEV in nM.

Plasma: Absolute concentrations of CoA in rat blood plasma ranged from 4.99 to 20.0 nM. The highest CoA values were also found in the blood plasma of healthy rats, where their level was 30–35% higher than that in the plasma of rats that were treated with TAA (Table 1, Figure 1A).

The concentration of acetyl-CoA in the blood plasma of rats was an order of magnitude lower and varied over the range 0.074–0.491 nM. In contrast to CoA, the plasma acetyl-CoA concentration in rats intoxicated with TAA was 25–50% higher than that of the control level (Table 1, Figure 1B). The greatest concentrations of CoA and acetyl-CoA in the blood plasma of rats 6 days after TAA administration were observed in animals that received relatively low or medium (200–400 mg/kg) doses of the toxin.

**Figure 2:**
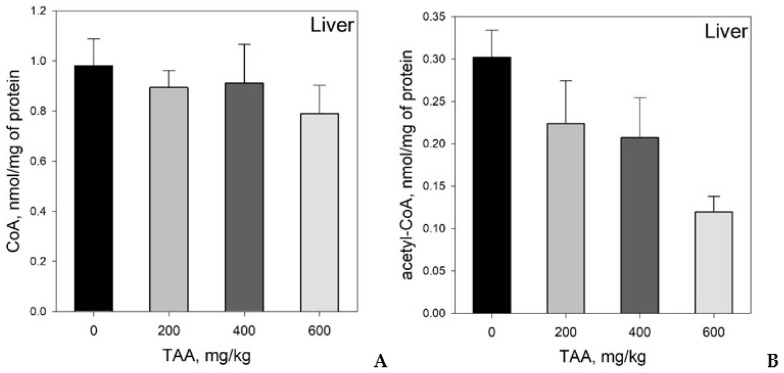
(**A**) Distribution of CoA and (**B**) acetyl-CoA in the livers of rats administered different doses of TAA. Data are given as AVG ± STDEV in nmol/mg protein.

Liver: Absolute concentrations of CoA in rat livers were in the range of 0.627–1.24 nmol/mg protein and were slightly lower (by 10–20%) in rats treated with TAA (Table 1, Figure 2A).

The levels of acetyl-CoA in rat liver were in the range of 0.100–0.350 nmol/mg protein (Table 1, Figure 2B). Moreover, these levels were also lower in the livers of TAA-treated animals (25–60%).

**Figure 3:**
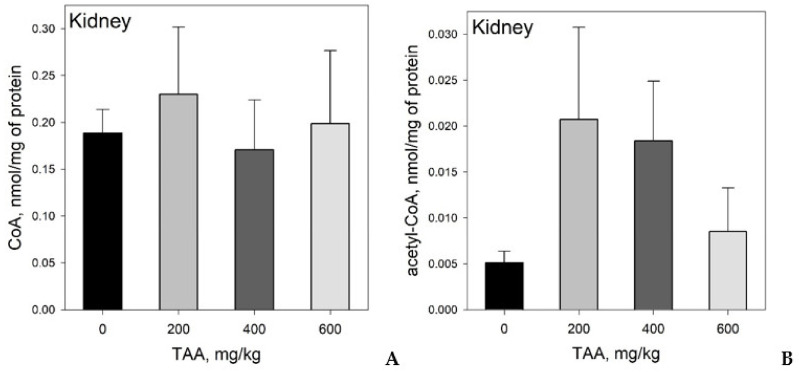
(**A**) Distribution of CoA and (**B**) acetyl-CoA in the kidneys of rats administered different doses of TAA. Data are given as AVG ± STDEV in nmol/mg protein.

Kidneys: CoA levels in rat kidneys ranged from 0.063–0.307 nmol/mg protein (Table 1, Figure 3A). The highest CoA values (20% higher than that of the control) were found in the group of rats treated with a low dose of toxin (200 mg/kg).

Acetyl-CoA levels ranged from 0.002 to 0.036 nmol/mg protein. The maximum level of acetyl-CoA (4-times higher than that of the control) also was found in the group of rats administered low or medium (200–400 mg/kg) doses of TAA (Table 1, Figure 3B).

**Figure 4:**
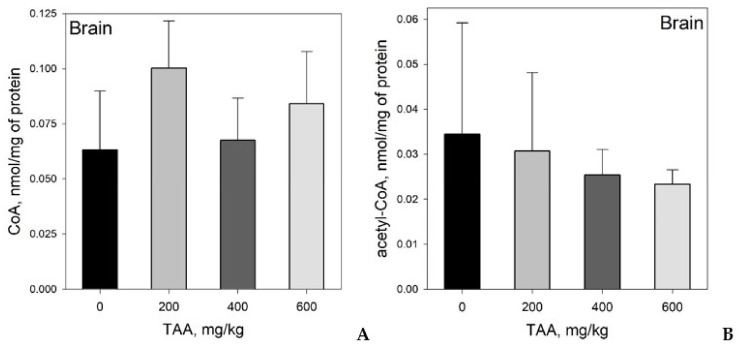
(**A**) Distribution of CoA and (**B**) acetyl-CoA in the brains of rats administered different doses of TAA. Data are given as AVG ± STDEV in nmol/mg protein.

Brain: CoA levels in rat brain tissues varied over the range of 0.018–0.132 nmol/mg protein, and maximum coenzyme values were also observed in the group of rats treated with a low dose of toxin (200 mg/kg). The increase in CoA in the brain tissues of this group of rats reached 60% relative to that of the control. In other groups of rats, the increase in CoA relative to the control was about 10–30% (Table 1, Figure 4A).

The concentrations of acetyl-CoA in the rat brain varied over the range of 0.014–0.057 nmol/mg of protein with a gradual decrease in the concentration of coenzyme from the control group of rats to the group receiving the highest (600 mg/kg) dose of TAA. The decrease in the levels of acetyl-CoA in the experimental groups, relative to the control, was 10, 25, and 35% (Table 1, Figure 4B).

**Figure 5:**
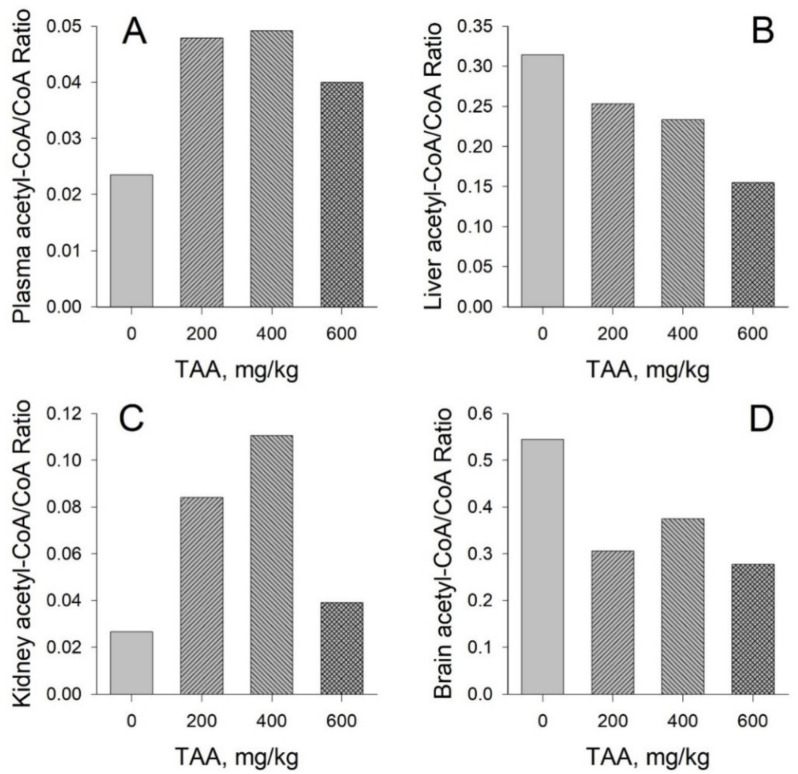
Ratios of acetyl-CoA/CoA in plasma (**A**), liver (**B**), kidney (**C**), and brain (**D**) of rats. The ratios are calculated from the corresponding AVG values for each tissue and plasma.

Acetyl-CoA/CoA ratio: The acetyl-CoA/CoA ratio calculated for plasma, liver, kidney, and rat brain was strikingly dose-dependent (Figure 5A–D).

In the plasma and kidney tissues of rats (Figure 5A,C), the change in the acetyl-CoA/CoA ratio depends on the dose of the administered toxin and is markedly different between liver and brain (Figure 5B,D). Thus, the data presented in Figure 5 show that the ratio of acetyl-CoA/CoA in kidney and plasma samples of TAA-treated rats is higher than that in the controls. However, this relationship is not linear. The highest ratio values were obtained for animals administered low and medium doses of TAA (200–400 mg/kg). However, in the group of animals administered a high dose of TAA (600 mg/kg), the acetyl-CoA/CoA ratio is lower than that in the groups administered low and medium doses, but higher than that in the control group. In the kidneys and blood plasma, the maximum difference in the acetyl-CoA/CoA ratios between the control group and the group of TAA-treated animals (400 mg/kg) was 0.03–0.11 for the kidneys and 0.019–0.050 for plasma.

In the livers and brains of the TAA-treated rats, as the dose of the toxin is increased, a progressive decrease in this ratio is observed (Figure 5B,D). In control samples of rat brain and liver, this ratio is 0.54 and 0.31. However, for rats that received the highest dose of TAA (600 mg/kg), it is half as much, 0.27 and 0.15, respectively.

## 4. Statistical Evaluation of the Data

The normal distribution of the concentrations of CoA and acetyl-CoA in each of the biological samples among the groups of rats was verified by the Shapiro–Wilk method with the FDR control procedure at the significance level *p* ≤ 0.05 by the Benjamini–Hochberg method, which led to 32 independent tests. Not a single refutation of the null hypothesis concerning the normality of distributions was received.

Testing for equality of the variances of the concentrations of CoA and acetyl-CoA in each of the types of biological samples between the studied groups led to eight independent tests (two metabolites in four species of samples). Based on the results of testing for normal distributions, checking for equality of variances was performed by the Bartlett method using the FDR control procedure at a significance level of *p* ≤ 0.05 by the Benjamini–Hochberg method. Not a single refutation of the null hypothesis was noted.

Next, we tested the hypothesis that the groups of rats do not differ in any of the metabolites in each of the tissue samples under consideration (the hypothesis that all groups are random samples from the same population) using the MANOVA method, where the influencing factor is the group, and the dependent variables are the observed concentrations of metabolites in each of the sample varieties. Thus, we had 16 individual subjects at our disposal, one influence factor with four levels of influence on eight variables (concentration of two metabolites in four varieties of samples) was chosen.

To test the hypothesis, the implementation of MANOVA in python from the “statsmodels.multivariate.manova” package was used. The *p* value results were obtained for four approaches to assessing the value of the F-criterion: (a) Wilks’ lambda, (b) Pillai’s trace, (c) Hotelling–Lawley trace, (d) Roy’s greatest root. Roy’s greatest root estimated *p* value = 0.0105; for both the Wilks’ lambda and Pillai’s trace approaches, the *p* value was < 0.02; and for Hotelling–Lawley trace the *p* value was = 0.116. It should be noted that Roy’s greatest root gives a lower bound for *p* value and is the least conservative of these approaches. In further tests, this method was used to cut off the obviously dead-end branches in the hierarchy of hypotheses while maintaining the maximum sensitivity of MANOVA to deviations in mean values in groups.

To answer the question: “If there are differences, for which tissues are they available?”, the hypothesis was tested for each of the four varieties of samples, that all groups are random samples from the same population using the FDR control procedure at a significance level of *p* ≤ 0.05 by the Benjamini–Hochberg method. According to the test results, in the future, we declined to consider the brain tissue, where the *p* value was equal to 0.3456. At the same time, the *p* values for blood plasma, livers, and kidneys were 0.0001, 0.0032, and 0.0063, respectively. All of these values were below the threshold *p* value = 0.0125, 0.025, and 0.0375, respectively, obtained by the Benjamini–Hochberg method to maintain the FDR at *p* ≤ 0.05 for four tests.

Assuming that in the remaining varieties of samples (blood plasma, liver, and kidney tissues) there are groups that are hidden by at least one of the CoA or acetyl-CoA metabolites, two Tukey’s HSD tests were used to isolate the outlier group for each of the sample varieties using the procedure FDR control at the significance level *p* ≤ 0.05 by the Benjamini–Hochberg method for two tests.

**Figure 6:**
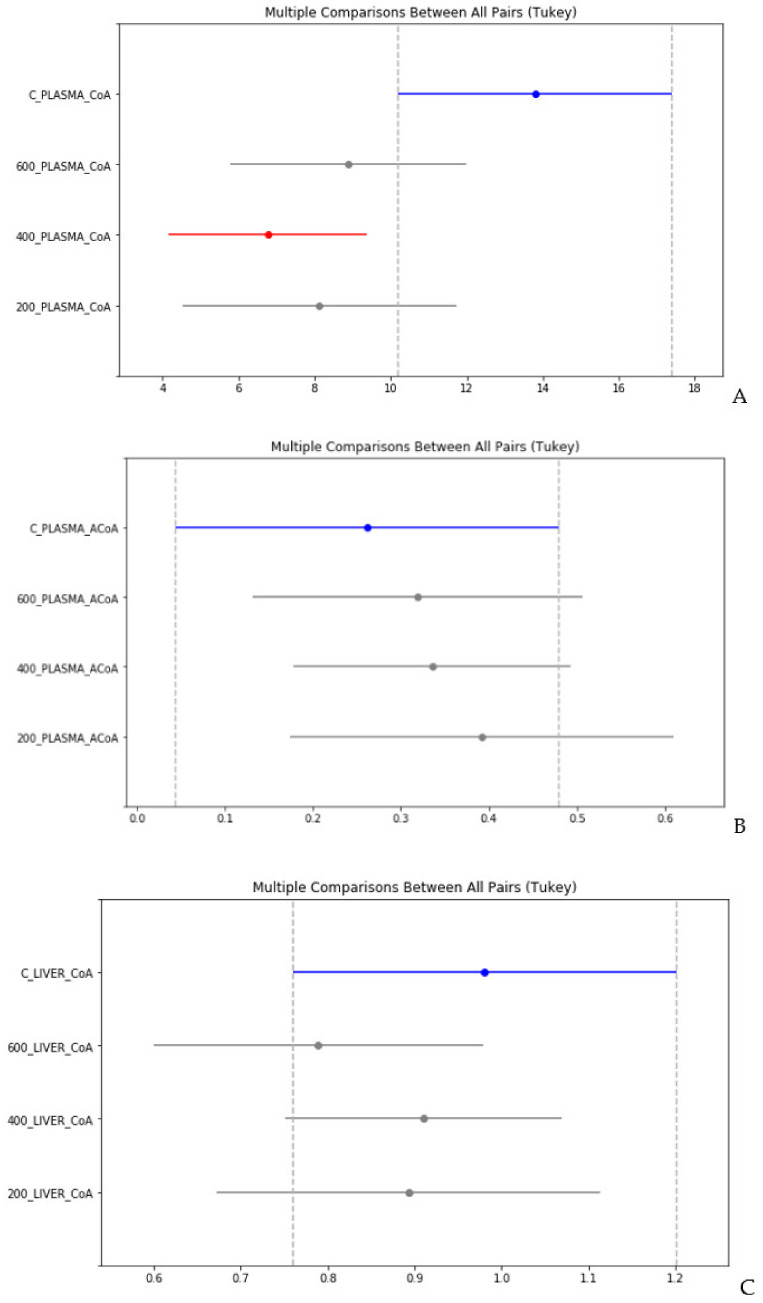

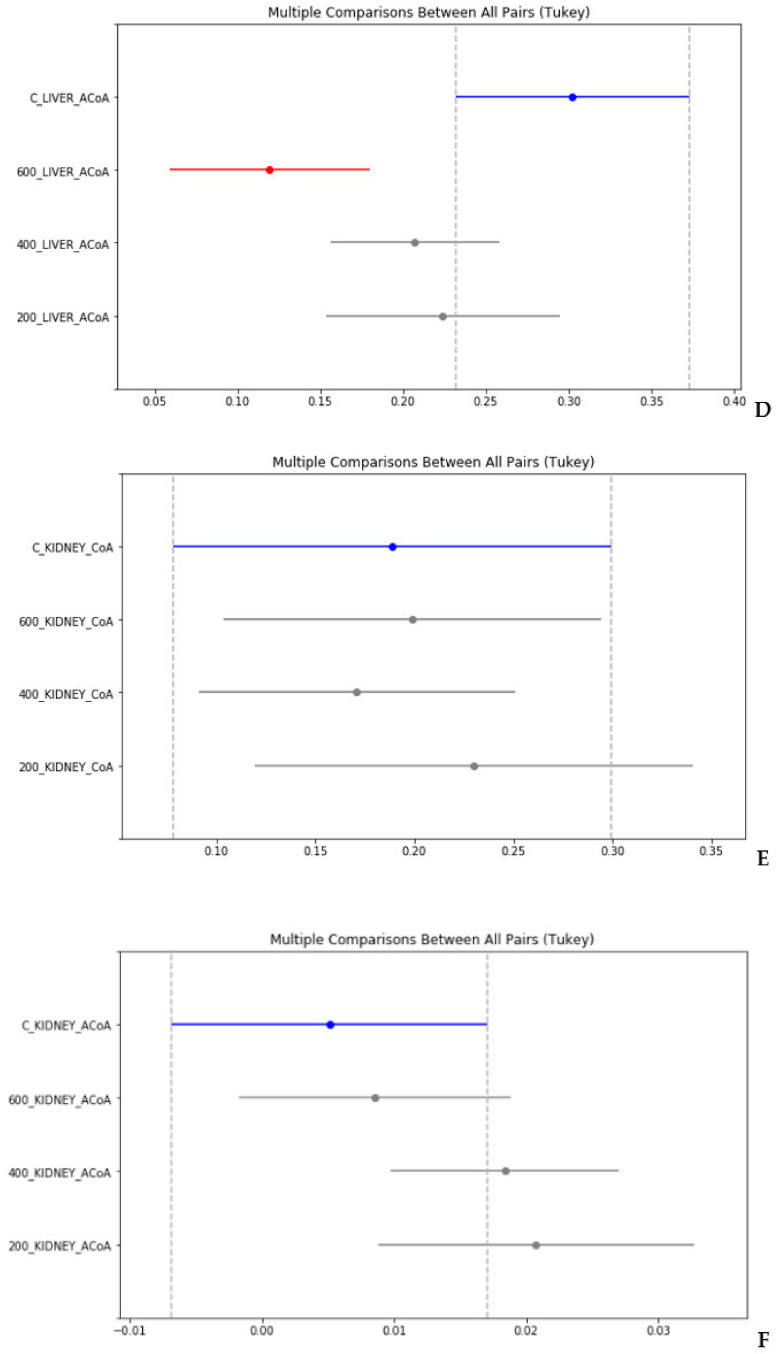
Multiple comparisons between all pairs (Tukey) for CoA and acetyl-CoA in plasma (**A** and **B**, respectively), in liver (**C** and **D**, respectively), and in kidney (**E** and **F**, respectively). C, control; 200, 400, 600, TAA concentration (mg/kg).

As a result, at a significance level of *p* ≤ 0.05, we found statistically significant differences in the concentration of CoA in the blood plasma for group 400 mg/kg and in the concentration of acetyl-CoA in the liver for group 600 mg/kg, compared to the corresponding controls (Figure 6A,D). Statistically significant differences in the concentrations of CoA and acetyl-CoA for plasma and all types of tissues between test groups 200, 400, and 600 mg/kg at this level of significance were not found (Figure 6B,C,E,F).

## 5. Discussion

### CoA and Acetyl CoA in Control and TAA-Treated Rats

The precursor of CoA in mammalian tissues is pantothenic acid. It is generally assumed that each tissue of the body is capable of independently taking up this acid into the cell and synthesizing CoA therein, although the intracellular localization of the requisite reactions remains unclear [13]. The concentrations of CoA in mitochondria and peroxisomes are higher than those in the cytosol and nucleus. CoA is distributed to subcellular organelles via dedicated transporters, most of which have been identified to be on the mitochondrial and peroxisomal membranes [10].

The role of CoA in the cell is multifaceted: The coenzyme acts as a carrier of the acyl groups, and at the same time it is a cofactor in many important metabolic processes, including the oxidation of fatty acids, carbohydrates, pyruvate, lactate, ketone bodies, and certain amino acids [1]. Thus, CoA is an essential coenzyme that is required for the normal production of metabolites and the functioning of the TCA cycle.

Its thioester, acetyl-CoA, produced during glycolysis, amino acid metabolism, and β-oxidation of fatty acids, is also an important participant in the TCA cycle and one of the central compounds that is involved in the regulation of metabolic homeostasis in the cell. The concentration of acetyl-CoA reflects the general energy state of the cell. Acetyl-CoA allosterically regulates several enzymes and affects the activity of a number of processes. This allosteric regulation, together with the acetylation/deacetylation ratio, contributes substantially to the balance between cellular catabolism and anabolism [8].

Basically, CoA participates mostly in anabolic processes in the cell, whereas acetyl-CoA participates mostly in catabolic processes aimed at breaking down large “building blocks” of the cell, such as complex carbohydrates, proteins, and lipids [40].

According to our calculated acetyl-CoA/CoA ratios for control and TAA-treated animals, the liver and brain exhibit similar trends in the recovery process after exposure to the toxin. This ratio in the TAA-treated animals is decreased by about 50% for the brain, but somewhat less so for the liver compared to the control values (Figure 5B,D). Different findings were observed for plasma and kidney. The acetyl-CoA/CoA ratio increased in the group of TAA-treated animals by about 2.5-fold in plasma and by about 3.5-fold in rat kidney (Figure 5A,C). An increase in this ratio may indicate the prevalence of apoptotic processes, which is also reflected in the ratio of both coenzymes [8].

Acetyl-CoA plays an important role in the activation of pyruvate carboxylase, as it is an obligate allosteric modifier of this enzyme [41]. When acetyl-CoA is deficient, a negligible amount of oxaloacetate is formed in tissues. Such a system of controls and balances in the cell helps regulate the biochemical transformations of the TCA cycle, so that there is usually no excess or lack of intermediate products. Imbalance occurs when, as a result of fat oxidation, excess acetyl-CoA accumulates in tissues (fasting), or when carbohydrate catabolism is not rapid enough to supply pyruvate, which is necessary for the formation of oxaloacetate (diabetes mellitus) [40,42,43].

Oxaloacetate has an important regulatory function in the TCA cycle. Depletion of tissue stores of oxaloacetate leads to a slowdown or termination of the entire cycle. The consequence can be catastrophic for the cell, not only because of the depletion of one of the most important energy sources, but also because in the absence of a sufficient amount of oxaloacetate (necessary for interaction with acetyl-CoA), an excess of acetoacetate and other “ketone bodies” appears in the tissues, leading to a condition known as ketosis [43].

The concentrations of both coenzymes in the cell very quickly respond to changes in the concentration of associated cellular metabolites, as well as to the effects of various external agents or stress [14]. Depending on the situation, the coenzymes can act as substrates or products, as well as allosteric or post-translational regulators of protein acetylation. For example, CoA and acetyl-CoA are important allosteric modulators of fatty acid and glucose oxidation, while the CoA derivative is a key regulator of the uptake and oxidation of fatty acids by mitochondria [44].

An analysis of energy metabolism using heart cells as an example indicates that CoA and its intermediates affect metabolic flows in different ways [44]. In recent years, special emphasis has been placed on the control of energy metabolic pathways by protein acetylation. In the human liver, almost every enzyme involved in glycolysis, fatty acid metabolism, TCA cycle, and urea cycle is acetylated [45]. This is due to the presence of a mechanism by which changes in the status of acetylation affect the enzymatic activity of the cell in response to a change in its needs for certain metabolites. In this regard, recent data on the regulation of acetylation of metabolic enzymes during oncogenesis open up new avenues in the search for potentially new treatments [46].

The formation of a pool of acetyl-CoA in a cell is based in part on the complex regulation of pyruvate concentrations. The formation of acetyl-CoA and its subsequent oxidation in the TCA cycle are influenced not only by the incorporation of two C atoms of pyruvate into the acetyl moiety of acetyl-CoA through the action of PDHC, but also by the reduction of a portion of the pyruvate pool to lactate through the action of LDH. In addition, pyruvate, through its carboxylation, can provide oxaloacetate for replenishment of intermediate compounds of the TCA cycle [47]. In general, PDHC activity plays an important role in the regulation of cellular energy metabolism in various tissues and organs, and in the selection of the optimal biosynthetic pathway. Upon reduction of the PDHC activity, pyruvate dehydrogenase phosphatase acts in opposition to PDK, the reactivation of which increases the flow of the acetyl moiety of acetyl-CoA into the TCA cycle.

As we have already mentioned, as shown in many studies, a single injection of TAA at a dose of 300 mg/kg in Wistar rats is sufficient to induce serious changes in the liver (e.g., [19]). 24 h after intraperitoneal administration of TAA (300 mg/kg), the levels of several marker enzymes indicative of liver damage were significantly increased in the serum, and an increase in total DNA fragmentation was observed, indicating the toxicity of this TAA dose to the liver [19,20]. Protein levels in rat tissue samples after TAA injection were also significantly reduced, which is probably due to the formation of protein adducts with TAA [19]. Nevertheless, after the administration of an even higher single dose of TAA, the process of complete liver regeneration was reported to begin within 72 h after the administration of the toxin [20].

A targeted therapeutic effect on cell metabolism, as well as on the control of metabolic fluxes in the cell, is challenging without knowledge of the concentrations of CoA and acetyl-CoA in tissues of various organs in healthy, pathological, and post-pathological states of the body.

Here we consider the change in the concentration of CoA and acetyl-CoA, as well as their concentrations in the tissues of various organs of rats after administration of a hepatotoxin. Samples of liver, kidney, brain, and blood plasma were collected and analyzed 6 days after administration of a single injection of TAA.

Plasma: Blood plasma connects all organs into a single system and helps maintain homeostasis at the level of the cell, organ, and the whole organism. Its main role is to supply nutrients to the cells of various organs and then transport waste products of cellular metabolism to the liver and kidneys for excretion from the body.

The distribution of CoA and acetyl-CoA (Figure 1A, B) in the plasma of TAA-administered rats indicates a persistent shift in the metabolism of animals even after nominal complete recovery of the liver from TAA exposure. We suggest that a decrease in the level of CoA (up to 50%) against the background of an increase in the concentration of acetyl-CoA (up to 50%) may reflect the presence of a shift in the functioning of the TCA cycle.

Liver: Compared to blood plasma, a notably different distribution of key metabolites was found in rat livers. Thus, the content of both coenzymes in the liver gradually and smoothly decreased (up to 20–60%) from that of the control group as the dose of TAA was increased. Moreover, the level of decrease in acetyl-CoA concentration outstripped the level of decrease in CoA. The decrease in the level of acetyl-CoA in the liver of rats after TAA intoxication may be a contributing factor to the acute phase of HE development.

A decrease in the concentration of liver CoA concomitant with an increase of succinyl-CoA and propionyl-CoA levels was observed in plasma of patients with fatal hepatotoxicity due to an overdose of valproate [47]. It was noted that a decrease in the liver content of CoA, which is critical for β-oxidation, can lead to a disruption of fatty acid metabolism [48].

Kidney: The distribution of both coenzymes in rat kidneys was significantly different from that in plasma and livers. It is interesting to note that the renal acetyl-CoA concentrations among the groups of TAA-induced animals was different compared to those of livers. A noticeable increase in the concentration of acetyl-CoA was observed with low and medium doses of TAA administration, while at a higher dose of TAA (600 mg/kg), its level decreased.

Brain: At an acute stage of HE, an increase in ammonia production is often accompanied by a decrease in pyruvate production, as a precursor of acetyl-CoA, and a sharp increase in lactate production [29,34,35]. An interesting study of the changes in lactate and pyruvate concentrations under the influence of ammonia was carried out on differentiated cerebrocortical astrocytic cells in primary culture [49]. The authors investigated the effect of ammonia on lactate and pyruvate production rates, and pyruvate/lactate ratios in these cells. Ammonia had almost no effect on the levels of these metabolites and their ratio if its concentration in the medium was relatively low (up to 1 mM of ammonia). However, when the ammonia level was increased to 3 mM, there was a significant increase in lactate production and a significant decrease in the pyruvate/lactate ratio. In undifferentiated cells, with an increase in the ammonia concentration to 3 mM, a significant increase (up to 80%) accumulation of lactate and inhibition of pyruvate accumulation (by 40%) occurred. Thus, the gradual accumulation of ammonia in the range of 1–3 mM led to a gradual decrease in the pyruvate/lactate ratio.

Kala and Hertz [49] also noted that the ammonia-induced decrease in the pyruvate/lactate ratio may be secondary to a decrease in cellular glutamate concentration, which may be related to increased glutamine production and possibly to subsequent malate/aspartate shuttle malfunction. This hypothesis was confirmed by the addition of glutamate to the incubation medium. This supplementation had no effect on the increase in lactate production, but significantly reduced the ammonia-induced decrease in the pyruvate/lactate ratio [49].

The neurons in the brain require large amounts of glucose to maintain their basic function. Neurons utilize 50–80% of the energy available to the adult human brain, and they use the oxidative metabolism of glucose as the main source of energy [50]. Pyruvate is the end product of oxidative glycolysis, and is the main sources of acetyl-CoA, a direct energy substrate in brain cells.

In some neurodegenerative conditions, PDHC activity is inhibited, resulting in decreased synthesis of acetyl-CoA in brain mitochondria. This, in turn, slows down the metabolic flow through the TCA cycle and causes an energy deficit and inhibition of a variety of synthesis reactions. Thus, a decrease in the level of acetyl-CoA in brain cells can significantly contribute to neurotoxic mechanisms and promote development of neurodegeneration [9].

It should be noted that brain tissues are very heterogeneous in structure and are distinguished by their complexity and compartmentation. Therefore, the measurement of acetyl-CoA concentrations in a homogenate of brain tissue can only be an average and does not take into account its content in individual compartments of the brain, where the distribution profile of acetyl-CoA may be quite heterogeneous. In general, the concentrations of acetyl-CoA in various subcellular compartments are low and vary over a moderately wide range under a variety of physiological and pathological conditions. These changes may serve as early primary signals that profoundly alter cell viability and function [9].

## 6. Conclusions

1. CoA and acetyl-CoA concentrations in various rat organs differ markedly. Thus, a decrease in CoA levels was observed in the order: Liver → kidney → brain, and acetyl-CoA levels in the order: Liver → brain → kidney.
2. Liver, kidney, brain, and blood plasma, in response to TAA exposure, are characterized by individual and unique patterns of distribution of CoA and acetyl-CoA compared to the physiological state. The patterns of these distributions may reflect the different metabolic roles of these organs.
3. The ratios of acetyl-CoA to CoA in the organs and blood plasma of healthy and TAA-treated rats reflect the balance between anabolic and catabolic processes in organs during the period of their active recovery from the toxic effect of TAA.
4. Despite an adequate recovery period after TAA intoxication, the concentrations of CoA and acetyl CoA in the rat liver and blood plasma, especially at higher doses of TAA, remained significantly reduced relative to the controls.

## Figures and Tables

**Table 1:** Concentrations of CoA and acetyl-CoA in plasma ^a^, liver ^b^, kidney ^b^, and brain ^b^ of control and TAA-treated rats.

TAA mg/kg	PLASMA		LIVER		KIDNEY		BRAIN	
	CoA	ACoA	CoA	ACoA	CoA	ACoA	CoA	ACoA
0	20.0	0.074	0.917	0.278	0.151	0.003	0.054	0.017
0	10.2	0.344	0.883	0.350	0.210	0.006	0.103	0.015
0	11.2	0.368	1.14	0.279	0.206	0.006	0.032	0.072
200	9.02	0.466	0.995	0.204	0.261	0.019	0.132	0.014
200	8.19	0.417	0.834	0.168	0.122	0.008	0.094	0.022
200	7.17	0.294	0.851	0.300	0.307	0.036	0.075	0.057
400	5.39	0.221	0.781	0.173	0.278	0.030	0.077	0.028
400	6.47	0.368	1.24	0.195	0.151	0.017	0.080	0.021
400	7.96	0.589	0.715	0.247	0.124	0.021	0.018	0.016
400	8.74	0.172	0.944	0.270	0.175	0.023	0.071	0.026
400	7.09	0.491	1.02	0.245	0.219	0.012	0.060	0.022
400	4.99	0.172	0.770	0.113	0.077	0.006	0.099	0.039
600	6.06	0.442	0.726	0.100	0.179	0.006	0.091	0.028
600	9.99	0.172	0.627	0.130	0.268	0.008	0.124	0.019
600	9.18	0.466	0.922	0.146	0.285	0.018	0.048	0.025
600	10.3	0.196	0.882	0.102	0.063	0.002	0.072	0.021

^a^ nM. ^b^ nmol/mg of protein; CoA, coenzyme A; ACoA, acetyl-CoA; TAA, thioacetamide.
